# Using multiple machine learning algorithms to classify elite and sub-elite goalkeepers in professional men’s football

**DOI:** 10.1038/s41598-021-01187-5

**Published:** 2021-11-22

**Authors:** Mikael Jamil, Ashwin Phatak, Saumya Mehta, Marco Beato, Daniel Memmert, Mark Connor

**Affiliations:** 1 School of Health and Sports Sciences, University of Suffolk, Ipswich, UK; 2 Institute of Training and Computer Science in Sport, German Sport University Cologne, Cologne, Germany; 3 Natural Computing Research and Applications Group, School of Business, University College Dublin, Dublin, Ireland

**Keywords:** Mathematics and computing, Computer science, Scientific data

## Abstract

This study applied multiple machine learning algorithms to classify the performance levels of professional goalkeepers (GK). Technical performances of GK’s competing in the elite divisions of England, Spain, Germany, and France were analysed in order to determine which factors distinguish elite GK’s from sub-elite GK’s. A total of (n = 14,671) player-match observations were analysed via multiple machine learning algorithms (MLA); Logistic Regressions (LR), Gradient Boosting Classifiers (GBC) and Random Forest Classifiers (RFC). The results revealed 15 common features across the three MLA’s pertaining to the actions of passing and distribution, distinguished goalkeepers performing at the elite level from those that do not. Specifically, short distribution, passing the ball successfully, receiving passes successfully, and keeping clean sheets were all revealed to be common traits of GK’s performing at the elite level. Moderate to high accuracy was reported across all the MLA’s for the training data, LR (0.7), RFC (0.82) and GBC (0.71) and testing data, LR (0.67), RFC (0.66) and GBC (0.66). Ultimately, the results discovered in this study suggest that a GK’s ability with their feet and not necessarily their hands are what distinguishes the elite GK’s from the sub-elite.

## Introduction

In the last decade, much research on football has been focussed on the identification of “key performance indicators”, hereafter referred to as KPI’s^1^. In sport KPI’s are defined as being factors that are more closely aligned with success for a specific team and individual^2^. Previous studies have been able to identify KPI’s in numerous sports including football, these identification procedures have tended to consist of subjective talent identification methods that rely heavily on the opinions of coaches and scouts^3^, or the use of a variety of traditional statistical techniques^4–11^.

Advancements in the methods and technologies used to track and measure match day player performance are rapidly increasing the amount of available data in sports^12^. Wearable technology^13^ and semi-automatic and automatic tracking systems^14,15^ are partly responsible for this surge in performance data available for analysis. Ultimately, this increase in data availability has allowed practitioners to move away from the historical reliance on the subjective opinions and instincts of experienced former professionals (with generally high error rates), towards more accurate and reliable statistical analysis^16^. Whereas in the past, the relative dearth of available sports data prohibited research in football^17^, advancements in data collection technologies have led to researchers facing the opposite problem where the sheer volume of data now available becomes an obstacle in itself, due to data processing becoming unmanageable^18^. It is due in part to the problem above that machine learning techniques are attracting more interest with regards to talent identification based research, as they can process large amounts of data and learn optimal model parameters from it^19^. Machine learning techniques could thus potentially provide coaches, analysts and players with additional information, which can be used to make crucial tactical decisions as well as more informed recruitment decisions at the highest level of elite football^20^.

In terms of identifying informative performance indicators, the position of goalkeeper (GK) in football has been frequently overlooked in previous research^21^. This is somewhat surprising, considering the goalkeeper is the most specialised position in a football team^22^ and their actions are considered to have a significant bearing on final match outcomes^23^. Rule changes such as the back-pass and the more recent 6-s release rule have necessitated the requirement for goalkeepers to have greater ball control and passing skills^24^. Modern day goalkeepers are often required to perform as ‘sweepers’ during defensive phases of play as well as be actively involved in the general build-up and attacking phases of play^24^. In a recently published systematic review of 70 Talent Identification focussed studies on football, the authors stressed how goalkeepers were frequently overlooked in their reviewed studies^24^.

MLA’s such as GBC and RF are capable of modelling non-linear relationships among dependent variables (DV’s) and the independent variables (IV’s) if such relationships exist in the mechanism of data creation (game of football)^25^. Previous studies report the existence of non-linear relationships between KPI’s and performance in most team sports^26^. However due to the highly parametrised nature of MLA’s and the various stochastic approaches used to optimize those parameters, different algorithms can produce different results when provided with the same dataset. The consequences of this behaviour can have real world implications and without dedicated ground truth data, it is difficult to decipher which MLA is the most appropriate choice to use when making informed decisions. To overcome the limitations of relying on a single model, multi-model approaches have been employed across a wide range of problem domains and industries^27^. One of the main advantages of using multiple models is the enhanced robustness they provide against variance and bias errors compared to a single model. Previous research has also demonstrated the performance benefits of using multiple models, specifically the ability of multiple weak models to outperform one strong model when they are combined^28^. In this study, we present a multiple model approach to classify elite goalkeepers from performance data and identify features, which distinguish them from their sub-elite counterparts. To the best of our knowledge, this multiple model approach has not been previously utilised for position specific Talent Identification purposes in football.

## Methods

### Data

Performance data specific to goalkeepers competing in several elite leagues across Europe over five seasons between the 2013/2014 and 2017/2018 seasons were obtained from Opta sports, renowned for their high degree of accuracy^11,29,30^. Specifically, the sample consisted of 353 GK’s that were performing throughout this 5-season period in the English Premier League, Spanish La Liga, French Ligue 1, and German Bundesliga. The data was pre-processed to remove constant (team ID, player ID, venue) and sparse features (goals scored, throw-ins taken) and refined further by incorporating KPI’s that have been previously identified as affecting a GK’s performance^1,21,23,31^. KPI’s of little or no relevance to this study (i.e. appearances, substitutions etc.) were removed. Ultimately, these procedures resulted in 73 unique features (KPI’s) and a total of 14,671 samples (a full list of extracted technical features is presented in the Appendix A-Table 5). The dataset was then balanced to obtain an equal number of classes by performing random under sampling resulting in a new dataset containing a total of 5918 samples for both classes combined (0 and 1).

### Research design

**Figure 1:**
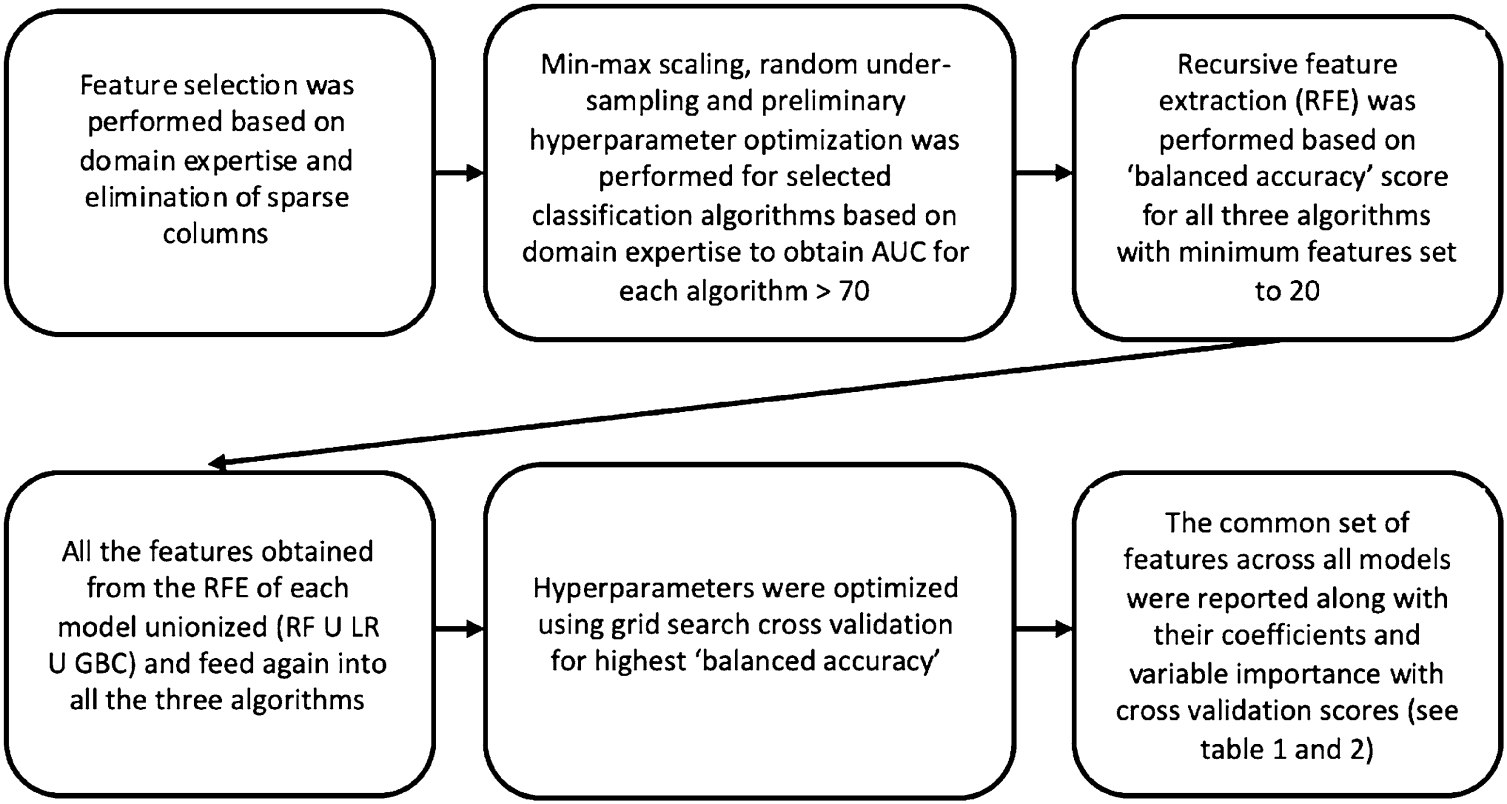
Machine learning pipeline for obtaining KPI’s.

Three different machine learning classification algorithms, Logistic Regression (LR), Random Forest Classifier (RFC), and Gradient Boosting Classifier (GBC) were used to classify goalkeepers who had played in the UEFA Champions League (UCL) (classified as: 1) as opposed to not having played in the UCL (classified as: 0). The UEFA Champions League, was purposely selected as the identifier of elite and sub-elite performance due to the competition being of the highest prestige^32^ and due to the fact this competition comprises of the very best teams and players^33^. For data balancing purposes, data for 53 non-UCL goalkeepers were excluded (random under sampling referred to above), resulting in a final sample of 300 GK’s. Data on UCL appearances were obtained from the increasingly popular Transfermarkt website^34,35^. Figure 1 outlines the machine learning pipeline used to conduct this study. Min–max scaling was performed and preliminary hyperparameter optimization was conducted for all three algorithms using the 73 filtered features to achieve a > 70 AUC (area under ROC curve) for each of the three models. Post optimization, recursive feature elimination was performed for all three classification algorithms using a ‘balanced accuracy’ scoring metric with the minimum allowable features set at 20^36^ to reduce the dimension of the problem space and only use the features providing the highest information gain. Post extraction of the features for each model was optimized for ‘balanced accuracy’ (average of the recall obtained on each class) using grid search cross validation^36^. The common features present in all three algorithms were reported with coefficients and variable importance. The pseudocode is presented in ESM Appendix A.

The coefficients from the LR provided both magnitude and direction of the effect, while the GBC and RFC provided feature importance scores. Ethical approval for this study was obtained by the ethics committee of the local institution. This study did not comprise of any testing on human subjects as all data utilised were secondary data obtained directly from Opta and full permissions to utilise this data for research purposes were obtained by all institutions involved in this study.

## Results

The results of 5-fold cross validation in Table 1 (training) and Table 2 (testing), show consistent accuracy, ROC - AUC (area under the receiver operating characteristic curve), and F1 scores, with the standard deviation being less than 5% for accuracy across all models. This suggests a ± 5% reliability and out of sample validity for all three models. LR has the highest accuracy for correct classification when evaluated on the testing data as compared to the other models (see confusion matrices in ESM Appendix 1). Both the GBC and RF tended to overfit on the training dataset, however, performance on the testing dataset was not compromised. Independent F-tests (using 50/50 cross validation) revealed significant differences between the three MLA’s utilised. Specifically, significant differences were discovered for F1 when comparing LR with RF (p = 0.042) and when comparing LR with GBC (p = 0.034). Significant differences were also discovered for accuracy when comparing LR with GBC (p = 0.032). In addition, significant differences were discovered for ROC - AUC when comparing LR with RF (p = 0.016) and when comparing LR with GBC (p = 0.011). The F statistics and their associated p-values are reported in Table 3.

**Table 1:** 5-fold cross validation results for training data (mean ± standard deviation).

	Logistic regression	Random forest classifier	Gradient boosting classifier
F1	0.70 ± 0.012	0.82 ± 0.005	0.71 ± 0.011
Accuracy	0.70 ± 0.014	0.82 ± 0.005	0.71 ± 0.011
ROC - AUC	0.77 ± 0.054	0.91 ± 0.003	0.78 ± 0.011

**Table 2:** 5-fold cross validation results for testing data set (mean ± standard deviation).

	Logistic regression	Random forest classifier	Gradient boosting classifier
F1	0.664 ± 0.055	0.64 ± 0.0723	0.651 ± 0.049
Accuracy	0.671 ± 0.0445	0.66 ± 0.045	0.66 ± 0.043
ROC - AUC	0.729 ± 0.057	0.723 ± 0.051	0.724 ± 0.049

**Table 3:** F test results.

Measure	Compared algorithms	F-statistic	p-value
F1	RF vs LR	5.159	0.042
	LR vs GBC	5.723	0.034
	RF vs GBC	1.503	0.342
Accuracy	RF vs LR	3.713	0.080
	LR vs GBC	5.906	0.032
	RF vs GBC	0.877	0.600
ROC - AUC	RF vs LR	8.159	0.016
	LR vs GBC	9.631	0.011
	RF vs GBC	1.149	0.467

Table 4 contains the set of common features reported by the three models post recursive feature elimination (15 in total). The results of the Logistic regression reveal features such as passes received (+ 3.39), % successful passes forwards (+ 1.16), GK short distribution (+ 0.81), and clean sheets (+ 0.34) were positively signed and important in distinguishing elite GK’s from sub-elite GK’s. The remaining features were revealed to be negatively signed and also distinguished elite GK’s from sub-elite GK’s; unsuccessful passes opposition half (− 0.5439), successful passes opposition half (− 0.5879), goals conceded (− 0.8896), GK long distribution (− 0.9598), touches (− 0.9882), total unsuccessful passes excluding crosses and corners (− 1.0458), successful passes final third (− 1.1860), GK pick up (− 1.3739), shots on conceded (− 1.4388), total successful passes excluding crosses and corners (− 1.6280) and successful long balls (− 2.6940). Successful passes in the opposition half (VI = 6.69%) were revealed to have the highest contributing factor for RFC and unsuccessful passes opposition half (VI = 7.03%) for GBC respectively.

**Table 4:** Feature importance from multiple machine learning algorithms.

Features	LR coefficients	RFC variable importance	GBC variable importance
Passes received	3.3866	0.0389	0.0395
% successful passes forwards	1.1582	0.0341	0.0404
GK short distribution	0.8093	0.0249	0.0255
Clean sheets	0.3488	0.0283	0.0218
Unsuccessful passes opposition half	− 0.5439	0.0499	0.0703
Successful passes opposition half	− 0.5879	0.0669	0.0537
Goals conceded	− 0.8896	0.0431	0.0390
GK long distribution	− 0.9598	0.0330	0.0327
Touches	− 0.9882	0.0321	0.0368
Total unsuccessful passes Excl crosses corners	− 1.0458	0.0457	0.0361
Successful passes final third	− 1.1860	0.0295	0.0290
GK—pick up	− 1.3739	0.0266	0.0253
Shots on conceded	− 1.4388	0.0302	0.0198
Total successful passes Excl crosses corners	− 1.6280	0.0303	0.0354
Successful long balls	− 2.6940	0.0627	0.0626

## Discussion

This study aimed to classify elite goalkeepers using performance data and identify features that distinguish them from their sub-elite counterparts using a robust multiple model machine learning approach. The results demonstrate that all MLA’s perform to a similar standard, with reasonable degrees of accuracy. The identification of a high number of common features among the three algorithms provides confidence that they are important in the separation of the elite from sub-elite goalkeepers. The inclusion, and relative performance, of the LR model, provides a suitable method of interpreting the feature importance scores further as the model can be reformulated to determine the changes in prediction accuracy when one of the features is changed by one unit.

Goalkeepers were categorised into elite (those performing in the UEFA Champions League) and sub-elite (those not performing in the UEFA Champions League) and many of the common features which distinguished between these two categories across all three machine learning algorithms were revealed to be passing based features as well as some ball distribution features. These results would suggest that it is not necessarily a goalkeeper’s ability with their hands that are their distinguishing attributes but their ability with their feet and thus their general football skills. However, it must be noted that playing styles commonly adopted in the UEFA Champions League (possession based)^37^ could also have potentially contributed to this particular finding.

Maintaining possession of the ball in football has been revealed in many studies as being a key determinant of team success^37,38^. As one way of maintaining possession includes executing successful passes, various aspects of the passing attribute such as accuracy, range, frequency, effectiveness and the longevity of passing sequences have been extensively reviewed^9,37,39–41^. Many studies that have focussed on passing have discovered those teams that present better values for variables such as “successful passes” can increase their opportunity to score goals, and thus win matches^38^. Evidence from this study distinguishes elite goalkeepers that are capable of successfully receiving passes, able to pass forward, and distribute the ball well (short) from their sub-elite counterparts.

Contrary to previous research revealing shot stopping and saves made as important key performance indicators for the position of goalkeeper^1,42^, the results of this study revealed no common features pertaining to these particular hand actions across the three MLA’s utilised. Many common features pertaining to other hand and foot actions concerning distribution however, were revealed in this analysis. A particularly pertinent finding of the present study is the positive effect of short distribution and the negative effect of long distribution revealed by the LR. This particular finding may be indicative of two things. Firstly, the differing playing styles between teams at the elite level, who tend to play a more technical game and those at a lower level who tend to play a more physical game^30,43–45^ and secondly, the evolving playing philosophy of modern day GK’s, which, consists of more short distribution around their own penalty areas^31^. Previous studies have reported that modern playing philosophies have evolved to include the goalkeeper more often with frequent passing activities^31^. Furthermore, Ref.^31^ discovered that goalkeepers used their feet to distribute the ball more often than their hands. At the time of their study, Ref.^31^ discovered that younger goalkeepers in their sample had more accurate kicking than their older counterparts suggesting coaching philosophies were already beginning to adapt. In addition, Ref.^31^ discovered further evidence of evolving playing philosophies as they discovered that younger goalkeepers played the ball to zones closer to the goal whereas older goalkeepers played the ball long more frequently in zones higher up the pitch. Ultimately, Ref.^31^ discovered that goalkeepers perform to better standards as the level of competition increases and thus their findings are in line with those discovered in this study. Previous research has also revealed that goalkeepers playing at the highest level are consistent with their distribution patterns, regardless of the game outcome, whereas goalkeepers performing at lower levels demonstrate differences in their choice of distribution and accuracy of distribution depending on the ongoing match status^23^, which could also partially explain the findings of this study. The results of the present study further re-enforce the findings of^23,31^ and imply that performance attributes pertaining to passing and distribution are key characteristics that distinguish between elite and sub-elite GK’s.

The present paper provides a suitable and robust method for identifying KPI’s from performance data which can be used for recruiting and talent identification purposes at both senior and youth levels. This research provides teams and recruiters with confidence that ML models can be used to classify talented players, thus saving them time and potentially assisting them in finding undervalued players in the market. Furthermore, these findings could potentially facilitate the adjustment of coaching philosophies moving forward, with GK’s increasingly being asked to be more involved in general build-up play^24^.

This study, however, was limited by several factors namely, the small number of MLA’s considered, the use of a single proxy measure of talent (technical) and some limitations in the dataset. Data on physical/psychological parameters were absent and the dataset did not comprise of advanced performance metrics (i.e. Expected Saves, xS), or information on the opponent’s shape/formation, or indeed the quality of passes received/distributed by GK’s. Future research should therefore look to incorporate physical/psychological performance data in combination with technical KPI’s to expand this area of research using a similar multiple machine learning approach with a wider range of MLA’s and proxy measures of talent. Future research may also consider applying similar methodologies to analyse the performances of outfield players in football or indeed other team sports. Furthermore, future researchers could consider alternative measures of elite and sub-elite performance (rather than the UCL vs non-UCL adopted in the present study).

## Conclusion

This study has discovered evidence that an elite goalkeeper’s ability with their feet and in particular their ability to pass the ball, is a distinguishing feature that separates them from sub-elite GK’s. Furthermore, an elite GK’s distribution ability was also revealed to be a distinguishing feature with short distribution having a positive effect and long distribution a negative effect. The method presented in the current study was shown to be accurate, robust and has the potential to be adapted to incorporate other variables such as market value, physical performance, and tactical requirements of the team. In addition, the findings of the present study have confirmed that the multiple MLA approach adopted in this study could be reliably utilised to aid recruitment, coaching and talent identification procedures in professional football.

## Supplementary Information

Supplementary Information.
