# Supplementary material for: Using multiple machine learning algorithms to classify elite and sub-elite goalkeepers in professional men’s football
Source: Sci Rep. 2021 Nov 22;11:22703. doi: 10.1038/s41598-021-01187-5 (PMC8609025; doi:10.1038/s41598-021-01187-5)
Supplement: Supplementary file 1 — Supplementary Information. [file 41598_2021_1187_MOESM1_ESM.docx]

**Appendix A**


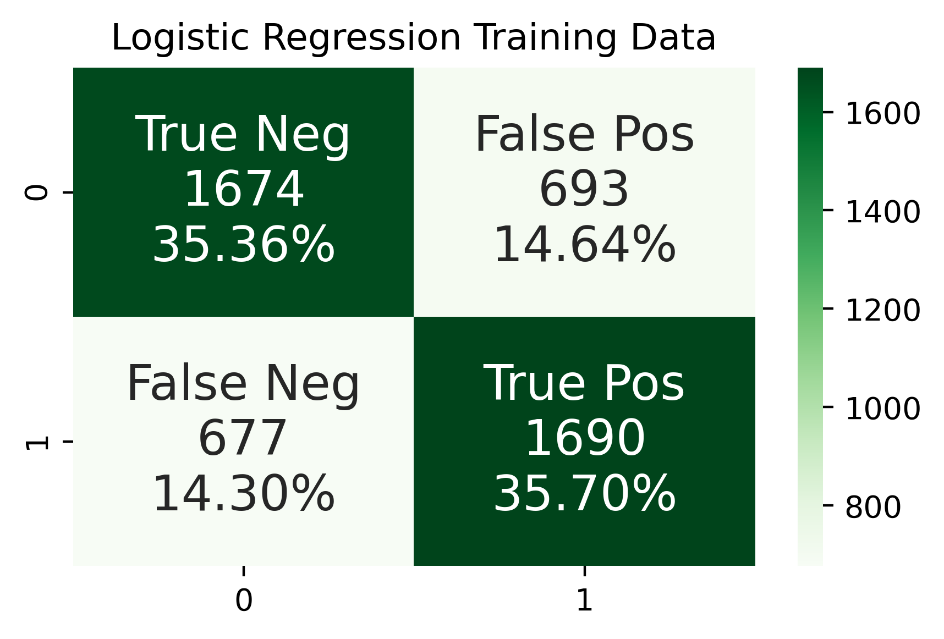


Supplementary Figure S1


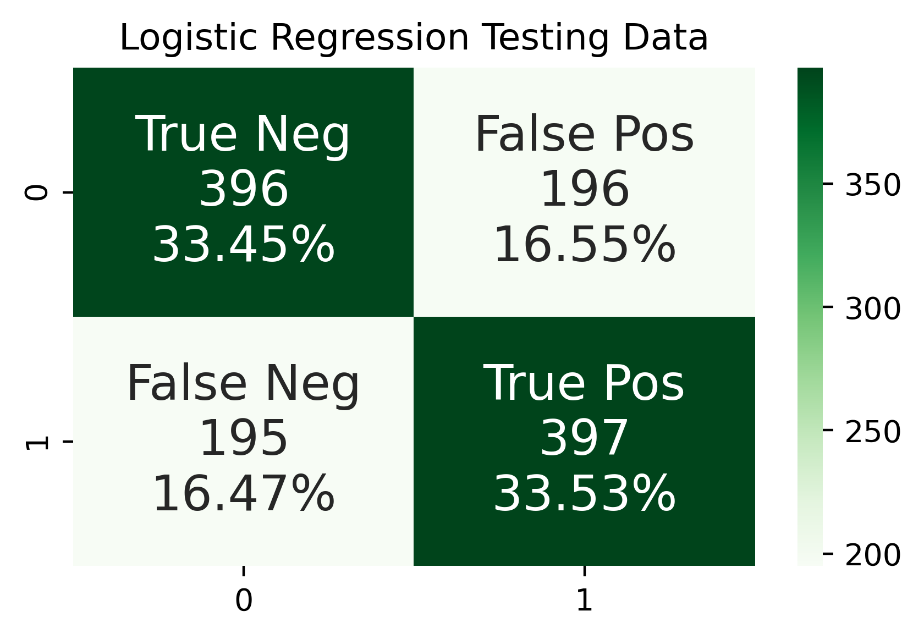


Supplementary Figure S2


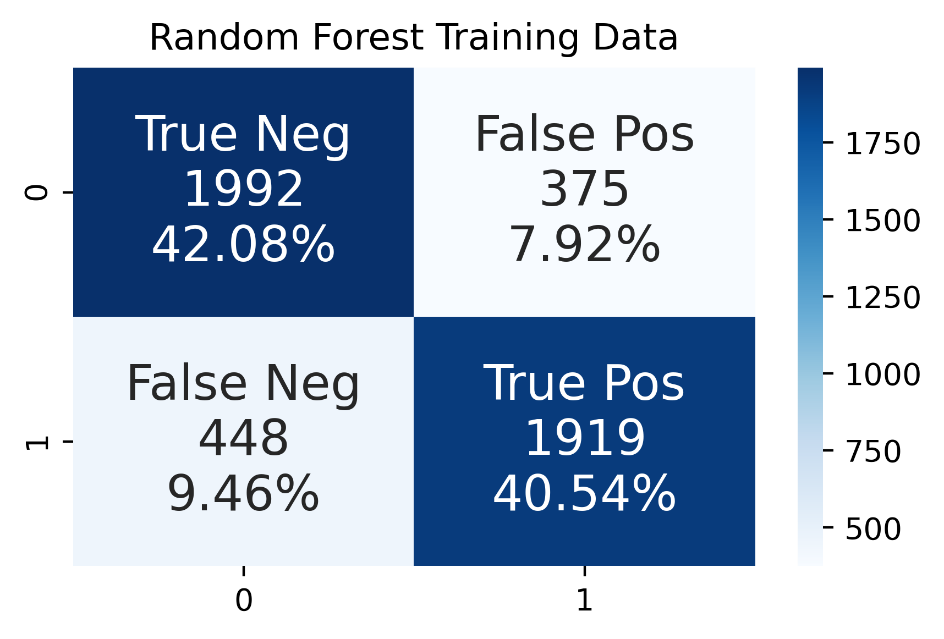


Supplementary Figure S3


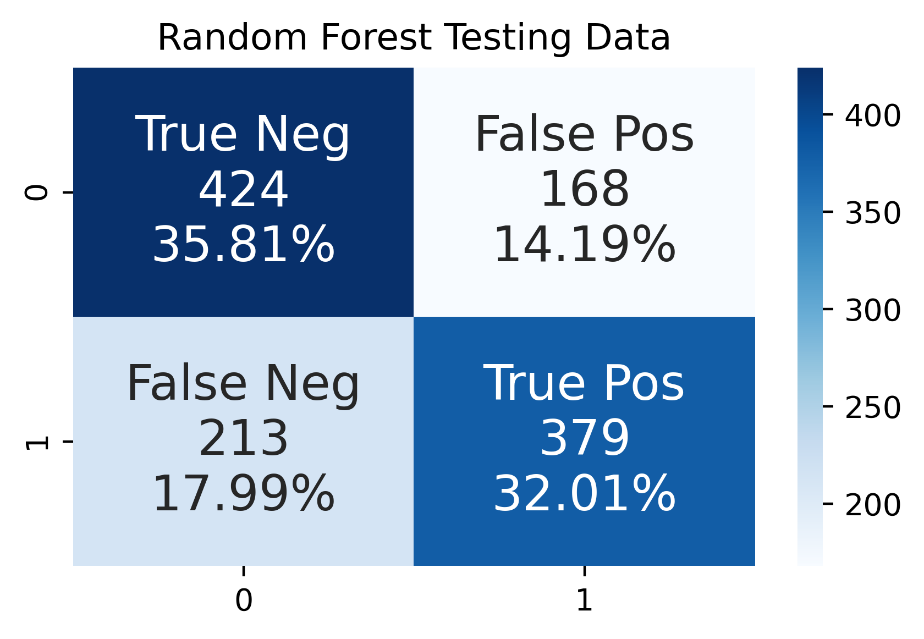


Supplementary Figure S4


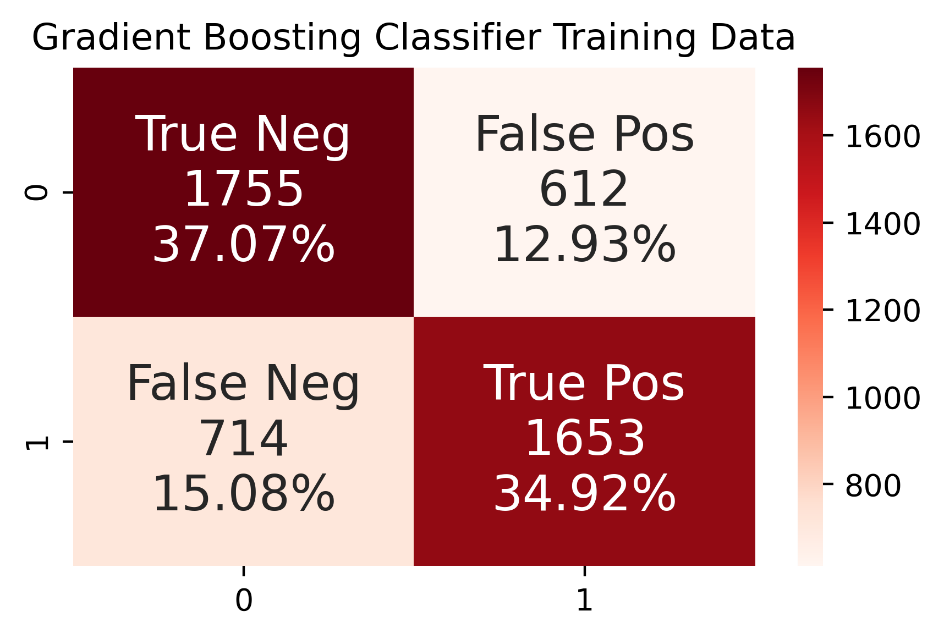


Supplementary Figure S5


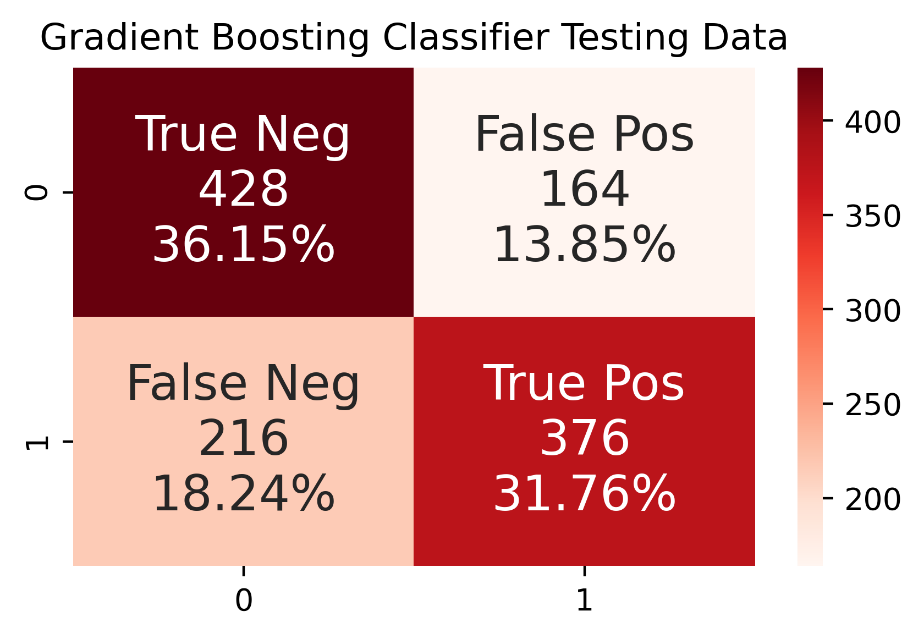


Supplementary Figure S6

**Pseudocode**

# **Pre-processing**

# Importing Data

# 14671 datapoints with 272 features

# Eliminating non GK related columns

# Eliminating columns with constant values

# Indexing Goalkeepers

# Champions league = 1

# non-champions league = 0

# Standardizing the Data between 1 and 0

# Balancing Data (equal number of Cl and NCL GKs) using Random Under sampling

# 🡪 5918 data points

# **Splitting the data**

# Stratified Train test split 80/20

**# Initial Algorithm Optimization**

# Optimize hyperparameters of Logistic regression & Random Forrest & Gradient Boosting Classifier

# Optimizing for auc_roc, f1 and accuracy to minimize the difference between train test results

**# Recursive Feature Extraction for min 20 features**

# Recursive feature extraction using LR, RF and GBC was run with min features set to 20

# Unionize extracted features from all three algorithms

# 🡪73 Features in total

**# Hyperparameter optimization for all 3 algorithms using RFE features**

# Logistic regression

# 5-fold Grid search Cross Validation to optimize f1, auc_roc, accuracy

# Resulting params

🡪 params = {‘C': 1.623776739188721, 'class weight': None, 'dual': False,'fit_intercept': True, ‘intercept_scaling': 1,

'l1_ratio': None, 'max_iter': 10000, ‘multi_class': 'auto' 'n_jobs': None, 'penalty': 'l2’, ‘random_state': 42,

'solver': 'liblinear', 'tol': 0.0001, 'verbose': 0,

'warm_start': False

}

# Random Forest

# 5-fold Grid search Cross Validation to optimize f1, auc_roc, accuracy

# Resulting params

🡪 params = {'bootstrap': True, ‘ccp_alpha': 0.0, 'class_weight': 'balanced',

'criterion': 'gini’, ‘max_depth': 10, 'max_features': 0.5, 'max_leaf_nodes': None, ‘max_samples': None, ‘min_impurity_decrease': 0.0, ‘min_impurity_split': None,

'min_samples_leaf': 10, ‘min_samples_split': 2, 'min_weight_fraction_leaf': 0.0, 'n_estimators': 1110,

'n_jobs': None, ‘oob_score': False, 'random_state': 42,

'verbose': 0, 'warm_start': False

}

# Gradient Boosting Classifier

# 5-fold Grid search Cross Validation to optimize f1, auc_roc, accuracy

# Resulting params

🡪 params = {‘ccp_alpha': 0.0, 'criterion': 'friedman_mse', 'init': None,

'learning_rate': 0.025, 'loss': 'deviance’, ‘max_depth': 5,

'max_features': 0.25, 'max_leaf_nodes': None, 'min_impurity_decrease': 0.0, 'min_impurity_split': None,

'min_samples_leaf': 100, ‘min_samples_split': 2,

'min_weight_fraction_leaf': 0.0, 'n_estimators': 500,

'n_iter_no_change': None, 'random_state': 42,

'subsample': 0.15, 'tol': 0.0001, 'validation_fraction': 0.1,

'verbose': 0, 'warm_start': False

}

**# Extracting Results**

# For each of the above algorithm

# Report features importance’s / Coefficients

# Report auc_roc curves (for train and test)

# 5-fold CV (f1, roc_auc, accuracy)

# Report Variable Importance for (LR ^ GBC ^ RF) (common features)

🡪 {'% successful passes forwards’, 'Clean Sheets', 'GK - Pick up',

'GK Long Distribution', 'GK Short Distribution', 'Goals Conceded',

'Passes Received', 'Shots on Conceded', 'Successful Long Balls',

'Successful Passes Final third’, 'Successful Passes Opposition Half',

'Total Successful Passes Excl Crosses Corners',

'Total Unsuccessful Passes Excl Crosses Corners', 'Touches',

'Unsuccessful Passes Opposition Half'

}

**# Statistical Testing**

# Conduct pairwise cross validated F-Test for comparing algorithm accuracy, roc_auc and f1 scores

|  |
| --- |
| **Table 4 – Extracted Features (technical)** |
|  |
| Champions League Performer, % successful passes forwards, Aerial Duels lost, Big Chances Faced, Challenge Lost, Clean Sheets, Corners Conceded, Crosses not Claimed, Defensive Aerial Duels Lost, Defensive Aerial Duels Won, Defensive Ground Duels Lost, Defensive Ground Duels Won, Duels lost, Error leading to Attempt, GK - Pick up, GK Distribution, GK Long Accuracy, GK Long Distribution, GK Short Accuracy, GK Short Distribution, GK Successful Distribution, GK Unsuccessful Distribution, Goals Conceded, Goals Conceded Inside Box, Ground Duels lost, Ground Duels won, Interceptions, Keeper Sweeper Unsuccessful, Key Set Pieces, Pass Forward, Pass Left, Pass Right, Passes Received, Penalties Conceded, Punches, Recoveries, Saves Body, Saves Caught, Saves Collected, Saves Feet, Saves Hands, Saves Made, Saves Made from Inside Box, Saves Parried Safe, Saves Stooping, Saves from Penalty, Shots On Conceded, Shots On Conceded Inside Box, Successful Ball Touch, Successful Dribbles, Successful Long Balls, Successful Long Passes, Successful Passes Defensive third, Successful Passes Final third, Successful Passes Middle third, Successful Passes Opposition Half, Successful Passes Own Half, Successful Short Passes, Total Blocked Shots Conceded, Total Fouls Conceded, Total Shots Conceded, Total Successful Passes All, Total Successful Passes Excl Crosses Corners, Total Unsuccessful Passes All, Total Unsuccessful Passes Excl Crosses Corners, Touches, Unsuccessful Ball Touch, Unsuccessful Long Balls, Unsuccessful Long Passes, Unsuccessful Passes Defensive third, Unsuccessful Passes Final third, Unsuccessful Passes Middle third, Unsuccessful Passes Opposition Half, Unsuccessful Short Passes |
|  |
